# Chemical Composition and Biological Activities of Endophytic Fungus *Talaromyces Wortmannii* LGT-4, Cultured in CYM Medium

**DOI:** 10.22037/ijpr.2020.1101051

**Published:** 2020

**Authors:** Zhong-Duo Yang, Yu Zhang, Wei-Hua Zhao, Zong-Mei Shu

**Affiliations:** *Department of Pharmaceutical Engineering, School of Life Science and Engineering, Lanzhou University of Technology, Lanzhou, China.*

**Keywords:** Endophytic fungus, Monoamine oxidase inhibitors, Chemical composition, Talaromyces wortmannii, Tripterygium wilfordi

## Abstract

In the present study, nine compounds (**1**-**9**) were isolated from *Talaromyces wortmannii *LGT-4 (an endophytic fungus from *Tripterygium wilfordi*) which was cultured in CYM Medium. Their structures were determined as 4-hydroxyphthalide (**1**), Fumitremorgin C (**2**), Ergosterol (**3**), 3-(2-hydroxypropyl)-8-hydroxy-3,4- dihydroisocoumarin (**4**), Cis-cyclo(*L*-Ala-*L*-Pro) (**5**), 6-Amino-3-(4-hydroxybenzyl)- 1,4-diazonane-2,5-dione (**6**), Aspergillumarin B (**7**), Deacetylisowortmin B (**8**), and Entonaemin A (**9**) based on NMR spectral data, as well as comparing with previous literature data. This is the first report of the isolation of compounds **1**-**2** and **4**-**7** from *Talaromyces* genus. All compounds were tested for their monoamine oxidase and phosphoinositide 3-kinase (PI3Kα) inhibitory activities. Compound **1**, **5** showed moderate anti-monoamine oxidase activity with IC_50_ value of 35 μg/mL, 28 μg/mL, respectively. Compound **9 **showed PI3K**α** inhibitory activity with IC_50 _value of 10.3 **μg**/mL.

## Introduction

Endophytes are microbes that colonise the internal tissues of plants without causing any overt negative effects immediately (1). They are proved to be a new source for natural compounds which have unique structure and wide range of biological activities, such as antitumor, antimicrobial, and antituberculosis (2). 

**Figure 1:**
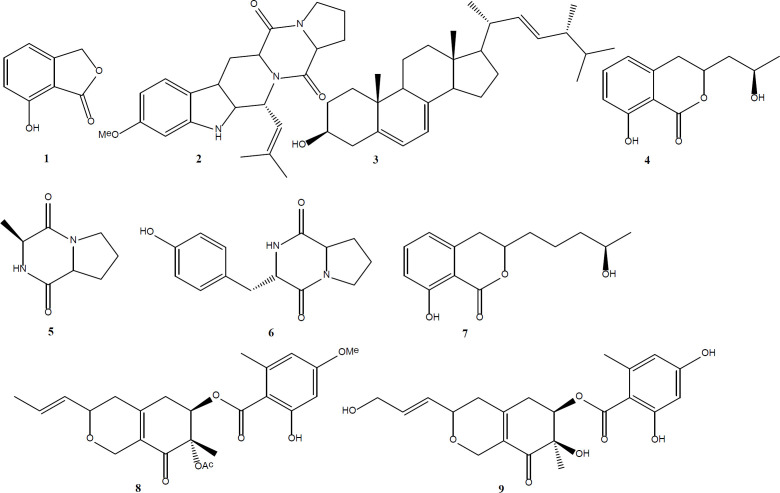
The structures of compounds **1**-**9**

During our research on endophytic fungi living in *Tripterygium wilfordii*, we isolated an endophytic fungi *Talaromyces wortmannii* LGT-4 (GenBank Accession No KF850714). In the past studies on this fungus, we have obtained a series of novel furanosteroid including secovironolide (3), wortmannines A-D (4), deacetylisowortmina A and B (5), wortmannolol (6), and wortmannine E (7) by changing culture mediums. Our further analysis work indicated that the altered fermentation media can really change its chemical profiles. In this paper, we reported another nine compounds (**1**-**9**) (Figure 1), which was obtained from the organic extract of CYM culture medium. All compounds were evaluated for their monoamine oxidase and phosphoinositide 3-kinase (PI3Kα) inhibitory activities.

## Experimental

Chemicals and Instrumentation: NMR spectra were obtained on a Bruker Avance-3 spectrometer at 600MHz. HPLC purification was performed using a Waters HPLC apparatus with two 510 pumps, one 2487 dual λ absorbance detector and a Phenomenex Gemini C18 column (10.0 mm × 250 mm, i.d., 5 μm). Silica gel (200-400 mesh) was from Qingdao Marine Chemical Co., Ltd. China. Macroporous resins (HPD-100) was purchased from Zhengzhou Qinshi Technology Co., Ltd., China. 

Fungus Material: *Talaromyces wortmannii* (*T. wortmannii) *LGT-4 (GenBank accession No. KF850714) was isolated from the healthy tissue of *Tripterygium wilfordi (T. wilfordi) *and identified as* T. wortmannii* based on both morphology on PDA and analysis of the DNA sequences of the ITS1-5.8S-ITS2 ribosomal RNA gene region. A GenBank search for DNA sequence similarity revealed that ITS1-5.8S-ITS2 of Lgt-4 was 99% homologous to that of *T. wortmannii* reference strains (GenBank No. FR667650).

Extraction and Isolation: The fungus LGT-4 was cultured in CYM liquid medium (for 1 L medium: including 12.0 g maltose, 2.0 g peptone, 2 g yeast extract, 0.244 g MgSO_4_, 0.460 g KH_2_PO_4_ and 1 L water) for 20d at 28 °C on a 50 L fermenter.

The fermentation broth was filtered, and then the filtrate was extracted with ethyl acetate twice. The ethyl acetate extract (5.0 g) was subjected to macroporous resin CC (eluting with 20, 40, 60, 80, and 100% EtOH) to yield five fractions (Fr. 1–5). Fr. 1 (1.1 g) was further separated by silica gel CC (CHCl_3_–MeOH, 50:1-1:1) to yield compound **1 **(2 mg) and six fractions (Fr. 1.1–1.5). Fr. 1.1 was further purified by semipreparative HPLC (MeOH–H_2_O, 45:55, 1.5 mL/min, 254 nm) to yield compound **2 **(t_R _= 21 min, 3 mg) and compound **4 **(t_R _= 45 min, 2 mg). Fr. 1.4 was purified by semipreparative HPLC (MeOH–H_2_O, 70:30, 1.1 mL/min, 254 nm) to yield compound **5 **(t_R _= 15 min, 2 mg) and compound **6 **(t_R _= 45 min, 2 mg). Fr. 2 (650 mg) was separated by silica gel CC (CHCl_3_–MeOH, 60:1-5:1) to yield compound **7 **(2 mg). Fr. 3 (350 mg) was separated by silica gel CC (Petroleum ether–acetone, 30:1-5:1) to yield compound **8 **(2 mg) and **9** (10 mg). Fr. 4 (440 mg) was separated by silica gel CC (Petroleum ether–acetone, 20:1) to yield compound **3** (2 mg).

Anti-MAO Bioassay: The procedure of testing MAO inhibiting activity was same with that reported in our previous paper (8).

PI3Kα Biochemical Assay: The procedure of testing PI3Kα inhibiting activity was the same with that reported in literature (9).

## Results and Discussion

This study focused on the compounds isolated from *Talaromyces wortmannii *LGT-4 (an endophytic fungus from *Tripterygium wilfordi*) which was cultured in CYM medium and evaluated biological activities. Nine compounds (**1**-**9**, Figure 1) were isolated. This is the first report of the isolation of compounds **1**-**2** and **4**-**7** from *Talaromyces* genus. Compound **1**, **5** showed moderate anti-monoamine oxidase activity with IC_50_ values of 35 μg/mL, 28 μg/mL, respectively. Compound **9 **showed PI3K**α **inhibitory activity with IC_50 _value of 10.3 **μg**/mL. 

*Structure elucidation of the isolated compounds:*

4-hydroxyphthalide (**1**):** 1** was obtained as a colorless needle. ^1^H-NMR of **1** showed 1, 2, 3- trisubstituted benzene ring signals (7.43 (1H, t, *J *= 7.9 Hz), 7.33 (1H, d, *J* = 7.9 Hz), 7.17 (1H, d, *J *= 7.9)). NMR of **1** also indicated that **1** had a OCH_2_ moiety (*δ*_H _5.30 (2H, s); *δ*_C _67.8) and ester carbonyl (*δ*_C _170.6). NMR data of **1 **was consistent with those of 4-hydroxyphthalide (10).

^1^H-NMR (600 MHz, CD_3_COCD_3_, δ, ppm, *J*/Hz): 7.43 (1H, t, *J *= 7.9 Hz, H-6), 7.33 (1H, d, *J* = 7.9 Hz, H-5), 7.17 (1H, d, *J *= 7.9 Hz, H-7), 5.30 (2H, s, H-9). ^13^C-NMR (150 MHz, CD_3_COCD_3_): 170.6 (C-2), 152.3 (C-3), 130.6 (C-5), 119.8 (C-6), 127.5 (C-7), 133.4 (C-8), 67.8 (C-9).

Fumitremorgin C (**2**): **2 **was obtained as a white amorphous powder. NMR of **2 **showed 1, 2,4- trisubstituted benzene ring signals (*δ*_H _7.43 (1H, d, *J *= 8.0), 6.86 (1H, s, H-19), 6.80 (1H, d, *J *= 8.0), a methoxyl signal (*δ*_H _3.83 (3H, s)), two amide carbonyl signals (*δ*_C_ 169.5, 165.7) and two methyl signals (*δ*_H _1.99 (3H, s), 1.68 (3H, s). NMR data of **2 **was consistent with those of fumitremorgin C (11).

^1^H-NMR (400 MHz, CDCl_3_, δ, ppm, *J*/Hz): 7.81 (1H, s, H-1), 7.43 (1H, d, *J *= 8.0, H-16), 6.86 (1H, s, H-19), 6.80 (1H, d, *J *= 8.0, H-17), 5.98 (1H, d, *J *= 9.2, H-3), 4.90 (1H, d, *J *= 9. 2, H-21), 4.18 (1H, dd,* J *= 11.6, 4.8, H-12), 4.11 (1H, t, *J *= 8.0, H-6), 3.83 (3H, s, OMe), 3.64 (2H, m, H-9), 3.50 (1H, dd,* J *= 16.0, 4.8, H-13a), 3.09 (1H, dd, *J *= 16.0, 11.6, H-13b), 2.38 (1H, m, H-7a), 2.25 (1H, m, H-7b), 2.06 (1H, m, H-8a), 1.99 (3H, s, H-24), 1.89 (1H, m, H-8b), 1.68 (3H, s, H-23); ^13^C-NMR (100 MHz, CDCl_3_): 169.5 (C-5), 165.7 (C-11), 156.6 (C-18), 137.0 (C-20), 133.9 (C-22), 132.2 (C-2), 124.2 (C-21), 120.7 (C-15), 118.8 (C-16), 109.5 (C-17), 106.3 (C-14), 95.3 (C-19), 59.2 (C-6), 56.8 (C-12), 55.7 (OMe), 51.0 (C-3), 45.4 (C-9), 28.6 (C-7), 25.7 (C-23), 23.0 (C-8), 21.9 (C-13), 18.1 (C-24).

Ergosterol (**3**): **3 **was obtained as a colorless crystal. NMR of **3 **showed six diagnostic steroid methyl signals (0.64 (3H, s), 0.80 (3H, d, *J *= 6.0), 0.83 (3H, d, *J *= 6.8 Hz), 0.90 (3H, d, *J *= 6.8 Hz), 0.94 (3H, s), 1.01 (3H, d, *J *= 6.8 Hz)), three double bond signals (*δ*_C _139.8, 119.6, 116.3, 141.3, 135.5, 132.0) and a oxygenated methine signal (*δ*_H_ 3.64 (1H, m, H-3); *δ*_C_70.4). NMR data of **3 **was consistent with those of ergosterol (12).

^1^H-NMR (400 MHz, CDCl_3_ , δ, ppm, *J*/Hz): 0.64(3H, s, H-18), 0.80 (3H, d, *J *= 6.0, H-26), 0.83 (3H, d, *J *= 6.8 Hz, H-27), 0.90 (3H, d, *J *= 6.8 Hz, H-21), 0.94 (3H, s, H-19), 1.01(3H, d, *J *= 6.8 Hz, H-4), 3.64 (1H, m, H-3), 5.15 (1H, m, H-22), 5.19 (1H, m, , H-23), 5.37 (1H,dd, *J *= 6.0, H-7), 5.56 (1H, d, *J *= 6.0, H-6); ^13^C-NMR (150 MHz, CDCl_3_): 38.3 (C-1), 32 .1 (C-2), 70.4 (C-3), 40.8 (C-4), 139.8 (C-5), 119.6 (C-6), 116.3 (C-7), 141.3 (C-8), 46.2 (C-9), 37.0 (C-10), 21.1 (C-11), 39.1(C-12), 42.7 (C-13), 54.5 (C-14), 23.0 (C-15), 28.2 (C-16), 55.7 (C-17), 12.0 (C-18), 16.3 (C-19), 40.4 (C-20), 21.1 (C- 21), 135.5 (C- 22), 132.0 (C- 23), 40.8 (C-24), 33.1 (C- 25), 19.9 (C-26), 19.6 (C-27), 17.6 (C-28).

3-(2-hydroxypropyl)-8-hydroxy-3, 4-dihydroisocoumarin (**4**):** 4 **was obtained as a white amorphous powder. NMR of **4 **showed isocoumarin signals (*δ*_H_ 6.69 (1H, d, *J* = 7.4 Hz), 7. 41 (1H, t, *J* = 7.1 Hz), 6.89 (1H, d, *J* = 7.4 Hz)) and two oxygenated methine signal (*δ*_H_ 4.63 (1H, m), 3.74 (1H, m)). NMR data of **4 **was consistent with those of 3-(2-hydroxypropyl)-8-hydroxy-3, 4-dihydroisocoumarin (13). 

 ESI-MS *m/z* 245.3 [M+Na]^+^. ^1^H-NMR (600 MHz, CDCl_3_, δ, ppm, *J*/Hz): 4.63 (1H, m, H-3), 2.95 (2H, m, H-4), 6.69 (1H, d, *J* = 7.4Hz, H-5), 7. 41 (1H, t, *J* = 7.1 Hz, H-6), 6.89 (1H, d, *J* = 7.4 Hz, H-7), 10.99 (1H, s, 8-OH), 1.90 (1H, m, H-9a), 1.77 (1H, m, H-9b), 3.74 (1H, m, H-12), 1.26 (3H, d, *J* = 6.5 Hz, H-13); ^13^C-NMR (150 MHz, CDCl_3_): 169.9 (C-1), 62.4 (C-3), 33.2 (C-4), 118.1 (C-5), 136.3 (C-6), 116.4 (C-7), 162.3 (C-8), 108.6 (C-9), 139.5 (C-10), 79.6 (C-12), 28.9 (C-13).

Cis-cyclo(*L*-Ala-*L*-Pro) (**5**): **5 **was obtained as a white amorphous powder. NMR of **5** indicated that **5** had a methyl (1.47 (3H, d, *J* = 6.8 Hz)) and a diketopiperazine unit (*δ*_H _4.13 (2H, m); *δ*_C _166.4, 51.3, 170.4, 59.4). NMR data of **5 **was consistent with those of cis-cyclo(*L*-Ala-*L*-Pro) (14).

^1^H-NMR (600 MHz, CDCl_3_, δ, ppm, *J*/Hz): 4.13 (2H, m, H-3, 6), 1.91 (1H, m, H-7a), 2.35 (1H, m, H-7b), 2.02 (1H, m, H-8a), 2.12 (1H, m, H-8b), 3.60 (2H, m, H- 9), 1.47 (3H, d, *J* =6.8Hz, H-10), 6.27 (1H, s, N-H); ^13^C-NMR (150 MHz, CDCl_3_): 166.4 (C-2), 51.3 (C-3), 170.4 (C-5), 59.4 (C-6), 28.3 (C-7), 22.9 (C-8), 45.6 (C-9), 16.2 (C-10).

6-Amino-3-(4-hydroxybenzyl)-1, 4-diazonane-2, 5-dione (**6**): **6** was obtained as a colorless needle. NMR of **6** indicated that **6** had a 1, 4- disubstituted phenyl ring (7.04 (2H, d, *J *= 8.0 Hz), 6.70 (2H, d, *J *= 8.0 Hz)) and a diketopiperazine unit (*δ*_H_ 4.35 (1H, t, *J *= 4.1 Hz), 4.04 (1H, dd, *J *= 6.2, 10.6 Hz); *δ*_C _166.9, 57.9, 170.7, 60.1). NMR data of **6 **was consistent with those of 6-amino-3-(4-hydroxybenzyl)-1, 4- diazonane-2, 5-dione (15).

^1^H-NMR (600 MHz, CD_3_OD, δ, ppm, *J*/Hz): 4.35 (1H, t, *J *= 4.1Hz, H-3), 4.04 (1H, dd, *J *= 6.2, 10.6 Hz, H-6), 2.08 (1H, m, H-7a), 1.20 (1H, m, H-7b), 1.79 (2H, m, H-8), 3.54 (1H, dt, *J *= 8.3, 11.9 Hz, H-9a), 3.35 (1H, dt, *J *= 6.9, 11.9 Hz, H-9b), 7.04 (2H, d, *J *= 8.0 Hz, H-2^’^, 6^’^), 6.70 (2H, d, *J *= 8.0 Hz, H-3^’^, 5^’^), 3.08 (1H, dd, *J *= 5.0, 14.0 Hz, H-7^’^a), 3.01 (1H, dd, *J *= 4.6, 14.0 Hz, H-7^’^b); ^13^C-NMR (150 MHz, CD_3_OD): 166.9 (C-2), 57.9 (C-3), 170.7 (C-5), 60.1 (C-6), 29.9 (C-7), 22.4 (C-8), 46.1 (C-9), 127.6 (C-1^’^), 132.1 (C-2^’^, 6^’^), 116.1 (C-3^’^, 5^’^), 157.7 (C-4^’^), 37.7 (C-7^’^).

Aspergillumarin B (**7**): **7 **was obtained as a colorless needle. ^1^H-NMR of **7 **showed isocoumarin signals (6.69 (1H, d, *J* = 7.4 Hz), 7. 41 (1H, t, *J* = 7. 7 Hz), 6.89 (1H, d, *J* = 8.4 Hz)), two oxygenated methine signal (*δ*_H_ 4.58 (1H, m), 3.84 (1H, m) and a methyl signal (1.21 (3H, d, *J* = 6.1 Hz)). ^1^H-NMR data of **7 **was consistent with those of aspergillumarin B (16).

ESI-MS *m/z* 273.4 [M+Na]^+^, 274.3 [M+Na+H]^+^. ^1^H-NMR (600 MHz, CDCl_3_, δ, ppm, *J*/Hz): 4.58 (1H, m, H-3), 2.95 (2H, m, H-4), 6.69 (1H, d, *J* = 7.4 Hz, H-5), 7. 41 (1H, t, *J* = 7. 7 Hz, H-6), 6.89 (1H, d, *J* = 8.4 Hz, H-7), 11.00 (1H, s, 8-OH), 1.90 (1H, m, H-1’a), 1.77 (1H, m, H-1’b), 1.61 (2H, m, H-2’), 1.51 (2H, m, H-3’), 3.84 (1H, m, H-4’), 1.21 (3H, d, *J* = 6.1 Hz, H-5’).

Deacetylisowortmin B (**8**): **8 **was obtained as a white amorphous powder. ^1^H-NMR of **8** indicated **8** had a 1, 2, 4, 6- tetrasubstituted phenyl ring (6.30 (1H, d, *J *= 2.4 Hz), 6.27 (1H, d, *J *= 2.7 Hz)), a methoxyl (3.78 (3H, s)), a methyl linked at the benzene ring (2.49 (3H, s)), an acetyl (2.07 (3H, s)) and a propenyl (5.54 (1H, dd, *J *= 15.4, 5.0 Hz), 5.81 (1H, dq, *J = *15.4, 6.5 Hz), 1.74 (3H, d, *J *= 6.5 Hz)). ^1^H-NMR data of **8 **was consistent with those of deacetylisowortmin B (4).

ESI-MS *m/z* 467.2 [M+Na]^+^. ^1^H-NMR (600 MHz, CDCl_3_, δ, ppm, *J*/Hz): 4.32 (1H, d, *J* = 16.7, H-1α), 4.59 (1H, d, *J* = 16.2, H-1β), 4.08 (1H, ddd, *J *= 10.1, 5.4, 5.0 Hz, H-3), 2.23 (1H, dd, *J *= 19.1, 5.4 Hz, H-4α), 2.35 (1H, dd, *J *= 18.4, 9.4 Hz, H-4β), 2.71 (1H, dd, *J *= 17.6, 6.1 Hz, H-5α), 2.57 (1H, m, H-5β), 6.20 (1H, dd, *J *= 10.5, 6.2 Hz, H-6), 5.54 (1H, dd, *J *= 15.4, 5.0 Hz, H-9), 5.81 (1H, dq, *J = *15.4, 6.5 Hz, H-10), 1.74 (3H, d, *J *= 6.5 Hz, H-11), 1.56 (3H, s, H-12), 2.07 (3H, s, H-2^’’^), 6.30 (1H, d, *J *= 2.4 Hz, H-3^’^), 6.27 (1H, d, *J *= 2.7 Hz, H-5^’^), 2.49 (3H, s, H-8^’^), 3.78 (3H, s, -OMe), 11.09 (1H, s, 2^’^-OH).

Entonaemin A (**9**): **9 **was obtained as a yellow oil. ^1^H-NMR of **9 **was similar with that of **8**. In the ^1^H-NMR spectrum of **9**, there are no methoxyl and acetyl signals, comparing with those of** 8**. A oxygenated methylene signal (4.22 (2H, brd, *J *= 5.0 Hz)) of **9** replaced the double bond methyl signal of **8 **(1.74 (3H, d, *J *= 6.5 Hz)), which showed that **9** had a propenol instead of propenyl unit. NMR data of **9 **was consistent with those of entonaemin A (17).

^1^H-NMR (600 MHz, CD_3_OD, δ, ppm, *J*/Hz): 5.01 (1H, d, *J *= 11.0 Hz, H-1a), 4.80 (1H, d, *J *= 11.0 Hz, H-1b), 5.53 (1H, s, H-4), 3.09 (1H, brd, *J *= 19.2 Hz, H-5a), 2.80 (1H, brd, *J *= 19.2 Hz, H-5b), 5.60 (1H, t, *J *= 3.2 Hz, H-6), 6.22 (1H, dt, *J *= 15.6, 1.6 Hz, H-10), 6.55 (1H, dt, *J *= 15.6, 5.0 Hz, H-11), 4.22 (2H, brd, *J *= 5.0 Hz, H-12), 1.44 (3H, s, H-9), 6.13 (2H, s, H-3^’^, 5^’^), 2.26 (3H, s, H-8^’^); ^13^C-NMR (150 MHz, CD_3_OD): 64.7 (C-1), 161.5 (C-3), 105.7 (C-4), 149.2 (C-4a), 32.9 (C-5), 78.3 (C-6), 75.5 (C-7), 197.0 (C-8), 115.5 (C-8a), 23.6 (C-9), 123.2 (C-10), 138.4 (C-11), 62.6 (C-12), 105.7 (C-1^’^), 166.3 (C-2^’^), 101.7 (C-3^’^), 164.0 (C-4^’^), 112.6 (C-5^’^), 144.9 (C-6^’^), 172.1 (C-7^’^), 24.5 (C-8^’^).

## References

[1] NagamV, NetalaVR, Desaraju S, Kalla C, Bobbu P, Dandu A, Tartte V (2014). Endophytes: a natural source for novel compounds. World J. Pharm. Res..

[2] Zhang HW, Song YC, Tan RX (2006). Biology and chemistry of endophytes. Nat. Prod. Rep..

[3] Ding HE, Yang ZD, Sheng L, Zhou SY, Li S, Yao XJ, Zhi KK, Wang YG, Zhang F (2015). Secovironolide, a novel furanosteroid scaffold with a five-membered B ring from the endophytic fungus Talaromyces wortmannii, lgt-4. Tetrahedron Lett..

[4] Fu GC, Yang ZD, Zhou SY, Yu HT, Zhang F, Yao XJ (2016). Two new compounds, deacetylisowortmins A and B, isolated from an endophytic fungus, Talaromyces wortmannii lgt-4. Nat. Prod. Res..

[5] Fu GC, Yang ZD, Zhou SY, Li XM, Yu HT, Yao XJ, Fang JG, Shu ZM, Xue HY, Wang YG (2016). Wortmannines A-C, three novel wortmannin derivatives with an unusual five-membered b ring from the endophytic fungus Talaromyces wortmannii, lgt-4. Tetrahedron Lett..

[6] Zhi KK, Yang ZD, Zhou SY, Yao XJ, Li S, Zhang F (2016). A new furanosteroid from Talaromyces sp a fungal endophyte isolated from Tripterygium wilfordii. Nat. Prod. Res..

[7] Sun JY, Yang ZD, Fu GC, Wang YG, Xue HY, Shu ZM (2017). A new wortmannine derivative from a Tripterygium wilfordii endophytic fungus Talaromyces wortmannii lgt-4. Nat. Prod. Res..

[8] Zhi KK, Yang ZD, Shi DF, Yao XJ, Wang MG (2014). Desmodeleganine, a new alkaloid from the leaves of Desmodium elegans as a potential monoamine oxidase inhibitor. Fitoterapia.

[9] Han FB, Lin SW, Liu P, Liu XJ, Tao J, Deng XB, Yi CQ, Xu H (2015). Discovery of a novel series of thienopyrimidine as highly potent and selective PI3K inhibitors. ACS Med. Chem. Lett..

[10] Guo LM, Shao CL, Liu X, Fang YC, Wei YX, Sun LL, Gu QQ, Zhu WM, Guan HS, Wang CY (2009). Chemical composition and antitumor activity of Sargassum pallidum. Chin. Tradit. Herbal Drugs..

[11] Ren H, Cao XL, Wang QE, Xu CM (2011). Antitumor metabolites from fungus Aspergillus sydomi D2-6. Chin. Pharm. J..

[12] Zhang YJ, Mills GL, Nari G (2002). Cyclooxygenase inhibitory and antioxidant compounds from the Mycelia of the Edible Mushroom Grifola frondosa. J. Agric. Food Chem..

[13] Zhao DQ, Wu ZH, Liu Dong, Pei YH, Lin WH, Bai J (2013). Secondary metabolites from marine sponge-associated fungus Hansfordia sp. J. Shenyang Pharm. Univ..

[14] Timo S, Thomas H (2005). Structures, sensory activity, and dose/response functions of 2,5-diketopiperazines in roasted cocoa nibs (Theobroma cacao). J. Agric. Food Chem..

[15] Xie XC, Mei WL, Zeng YB, Lin HP, Zhuang L, Dai HF, Hong K (2008). Cytotoxic constituents from marine actinomycete Streptomyces sp124092. Chem. J. Chin. Univ..

[16] Qi J, Shao CL, Li ZY, Gan LS, Fu XM, Bian WT, Zhao HY, Wang CY (2013). Isocoumarin derivatives and benzofurans from a sponge-derived Penicillium sp. Fungus. J. Nat. Prod..

[17] Li LQ, Yang YG, Zeng Y, Zou C, Zhao PJ (2010). A new azaphilone, kasanosin C, from an endophytic Talaromyces sp. T1BF. Molecules.

